# Geranylgeranyl diphosphate synthase: Role in human health, disease and potential therapeutic target

**DOI:** 10.1002/ctm2.1167

**Published:** 2023-01-17

**Authors:** Molly E. Muehlebach, Sarah A. Holstein

**Affiliations:** ^1^ Cancer Research Doctoral Program University of Nebraska Medical Center Omaha Nebraska USA; ^2^ Department of Internal Medicine University of Nebraska Medical Center Omaha Nebraska USA

**Keywords:** geranylgeranyl diphosphate synthase, geranylgeranylation, isoprenoid biosynthesis pathway, small GTPases

## Abstract

Geranylgeranyl diphosphate synthase (GGDPS), an enzyme in the isoprenoid biosynthesis pathway, is responsible for the production of geranylgeranyl pyrophosphate (GGPP). GGPP serves as a substrate for the post‐translational modification (geranylgeranylation) of proteins, including those belonging to the Ras superfamily of small GTPases. These proteins play key roles in signalling pathways, cytoskeletal regulation and intracellular transport, and in the absence of the prenylation modification, cannot properly localise and function. Aberrant expression of GGDPS has been implicated in various human pathologies, including liver disease, type 2 diabetes, pulmonary disease and malignancy. Thus, this enzyme is of particular interest from a therapeutic perspective. Here, we review the physiological function of GGDPS as well as its role in pathophysiological processes. We discuss the current GGDPS inhibitors under development and the therapeutic implications of targeting this enzyme.

## Graphical abstract

- Geranylgeranyl diphosphate synthase (GGDPS), an enzyme in the isoprenoid biosynthesis pathway, is responsible for the production of geranylgeranyl pyrophosphate (GGPP) used for protein geranylgeranylation.
- Dysregulation of GGDPS expression and/or activity is implicated in the pathophysiology of type 2 diabetes, liver disease, pulmonary disease and several malignancies.
- GGDPS inhibitors have promising efficacy in preclinical models of malignancy and pulmonary fibrosis.

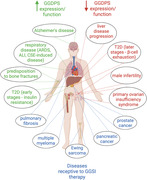

## 1 INTRODUCTION

**FIGURE 1:**
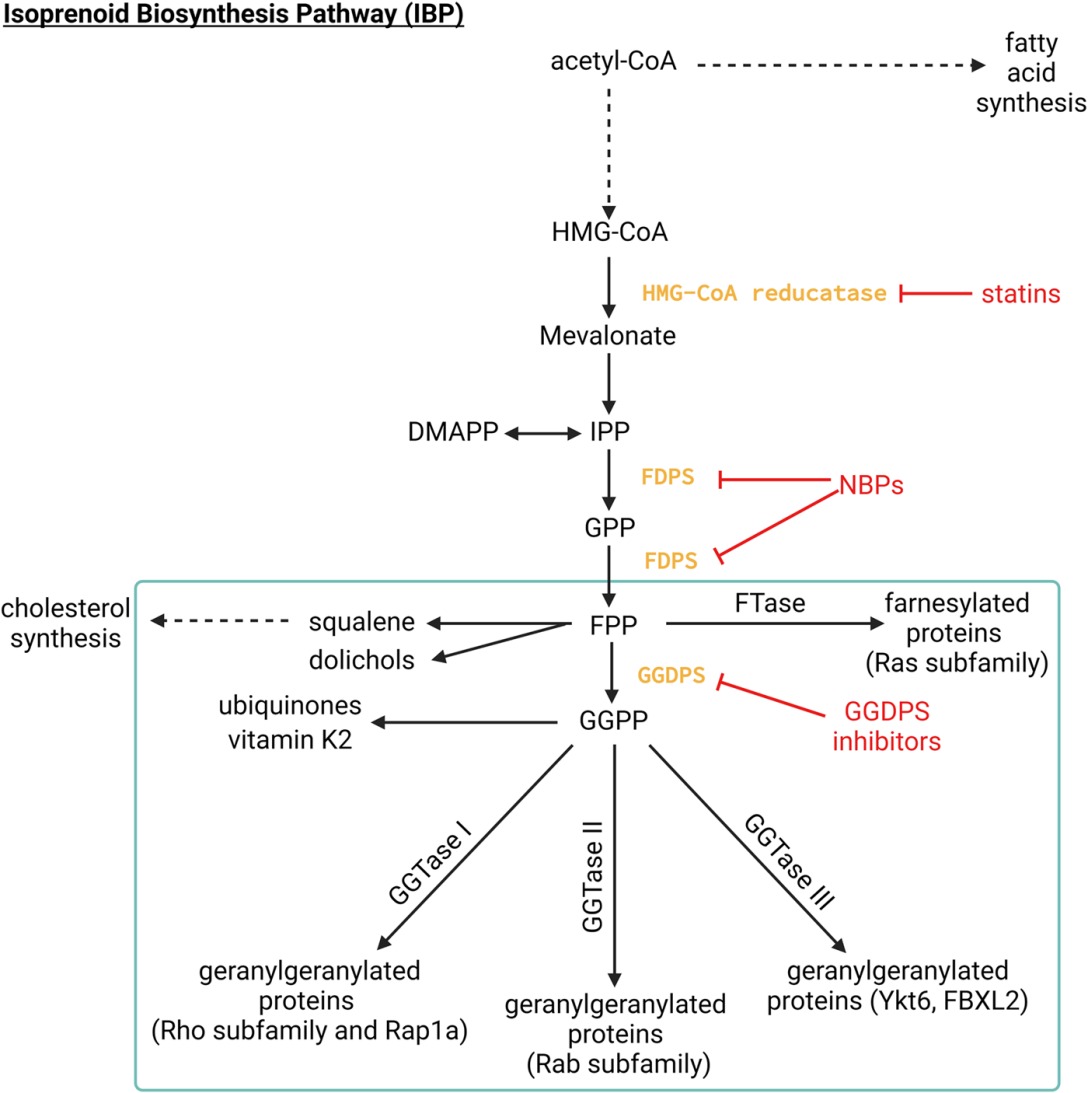
The mammalian isoprenoid biosynthesis pathway. Inhibitors (shown in red) include statins used most commonly for treatment of hypercholesterolemia, nitrogenous bisphosphonates (NBPs) used for treatment of various bone diseases and GGDPS inhibitors which have not yet been approved for clinical use.

Geranylgeranyl diphosphate synthase (GGDPS) is an essential enzyme in the isoprenoid biosynthesis pathway (IBP) (Figure 1). It facilitates the production of the 20‐carbon isoprenoid geranylgeranyl pyrophosphate (GGPP), which acts as a substrate for the post‐translational modification of proteins (geranylgeranylation) as well as a precursor of vitamin K2 and ubiquinone. Protein prenylation enables proper localisation, and thus function, of proteins that play key roles in signalling pathways, cytoskeletal regulation and intracellular transport. While other reviews have focused on topics such as inhibitors of the IBP and protein prenylation, less attention has been paid to the key roles of GGDPS in human health and disease and the implications this has for the implementation of novel therapeutic strategies.

The present review provides a comprehensive overview and synthesis of the current literature examining the role of GGDPS in human disease. This review takes a mechanistic approach, exploring the role of GGDPS in modulating disease processes such as insulin resistance and development of type 2 diabetes (T2D), liver disease, pulmonary disease and others. Also explored are the pharmacological agents that impact GGDPS activity, as these agents have not only provided insight into the sequelae of disrupting GGDPS activity, but also have potential therapeutic relevance for a variety of malignancies and other human diseases.

## 2 THE IBP

The IBP, also referred to as the mevalonate pathway, is responsible for the production of all mammalian isoprenoids (Figure 1). The pathway begins in the endoplasmic reticulum (ER) with the rate‐limiting conversion of 3‐hydroxy‐3‐methylglutaryl‐coenzyme A (HMG‐CoA) to mevalonate via the enzyme HMG‐CoA reductase (HMGCR). Through further phosphorylation and decarboxylation, mevalonate is converted to isopentenyl pyrophosphate (IPP). IPP and its isomer dimethylallyl pyrophosphate (DMAPP) then undergo subsequent condensation reactions via farnesyl diphosphate synthase (FDPS) to form first the 10‐carbon geranyl pyrophosphate (GPP) and then the 15‐carbon farnesyl pyrophosphate (FPP). FPP can then be utilised in the ER for sterol and dolichol synthesis. In the cytosol, the enzyme GGDPS utilises FPP along with IPP to catalyse the formation of the 20‐carbon GGPP. GGPP can also be used for other purposes, such as the synthesis of vitamin K2 and ubiquinone which is utilised during oxidative phosphorylation.^1^, ^2^ Finally, following production of mammalian isoprenoids FPP and GGPP, prenyltransferases, such as farnesyl transferase (FTase) and geranylgeranyl transferases (GGTase I, GGTase II and GGTase III), catalyse the addition of the isoprenoids to target proteins.^3^, ^4^, ^5^ This post‐translational modification is known as protein prenylation.

Prenyltransferases catalyse the addition of isoprenoids FPP or GGPP to a cysteine residue near the C‐terminus of the target substrate. Substrates for prenylation include Ras, Rho and Rab families of small GTPases, nuclear lamins, as well as certain kinases and phosphatases. It is the motif encompassing the C‐terminal cysteine residue that confers substrate specificity for prenyltransferase enzymes.^6^ For FTase and GGTase I, the cysteine is found in the consensus sequence referred to as the CAAX box, in which case the C refers to the Cys residue while A represents an aliphatic residue. Variation amongst the identity of the X residue is what confers specificity for either FTase or GGTase I: FTase targets substrates with a Met, Ser, Gln, Ala or Cys while GGTase I targets those with a Leu or Glu.^7^ However, these enzymes are not mutually exclusive with regards to substrate specificity. K‐Ras and N‐Ras, which are usually farnesylated, can be geranylgeranylated by GGTase I when FTase is inhibited.^8^, ^9^ Similarly, RhoB has been identified as a substrate of both FTase and GGTase I.^7^ This crossover may be due to the fact that FTase and GGTase I share a common α‐subunit.^10^

GGTase II, also referred to as Rab GGTase because of its specificity for small GTPases of the Rab sub‐family, targets a different recognition motif including XXCC, XCXC, CCXX, CCXXX or CXXX with the X residue varying depending on the substrate. Similar to FTase and GGTase I, the enzyme is a heterodimer. However, its α‐subunit only has 27% identity with that of the FTase/GGTase I α‐subunit, while the β‐subunit shows 29% identity to FTase.^11^ GGTase II also differs from FTase/GGTase I in that it requires the assistance of the Rab escort protein (REP1) which recruits substrate proteins to the enzyme, binds to GGTase II and facilitates the trafficking of the Rab proteins post‐prenylation.^12^, ^13^ In addition, GGTase II substrate targets are often doubly geranylgeranylated.

GGTase III is a recently discovered prenyltransferase enzyme that catalyses the double prenylation of the ubiquitin ligase FBXL2, as well as Golgi SNARE^14^ protein Ykt6 in combination with FTase.^5^, ^15^ Similar to GGTase II, GGTase III requires a chaperone protein (SKP1) for geranylgeranylation. It has been reported to share an identical β‐subunit with GGTase II but has a distinct α‐subunit.

Discovering the identity and function of prenylated proteins remains an active area of investigation. Use of GGPP probes and alkynyl C15 pyrophosphate derivatives through metabolic labelling have enabled identification of substrates of the prenylome.^6^ One study discovered 80 substrates, 64 of which were identified for the first time at an endogenous expression level.^16^ Further clarification of the prenylome will permit identification of potential targets of therapies that disrupt protein prenylation.

## 3 GGDPS STRUCTURE

**FIGURE 2:**
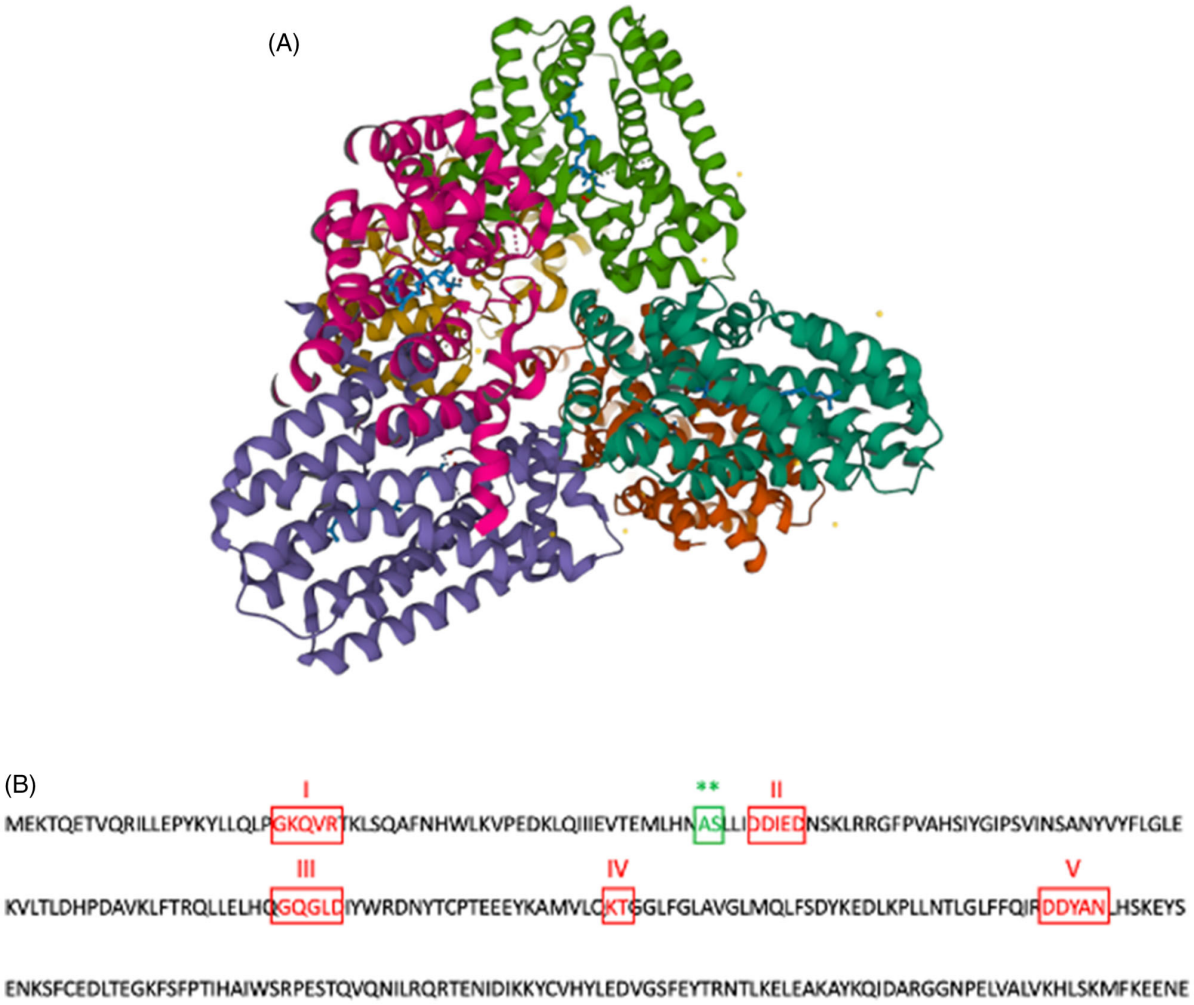
Human GGDPS. (A) Protein structure of human GGDPS with bound GGPP (blue), water molecules (yellow) and Mg^2+^ ions (red). (B) Amino acid sequence for human GGDPS. Conserved sequence motifs are shown in red and labelled (I–V). Residues unique to GGDPS (not present in FDPS) that allow for chain length elongation are shown in green. Images created from the RCSB Protein Data Bank (www.rcsb.org) of PDB Ihttps://doi.org/10.2210/pdb2Q80/pdb (Kavanagh et al.^17^).

Human GGDPS was first characterised by Kavanagh et al. in 2006.^17^ Characterisation of its paralogue FDPS as well as orthologues in species such as *S. cerevisiae*, *T. thermophilus*, *P. horikoshii* and *B. taurus*, have provided further structure‐function information. Kavanagh et al. reported that GGDPS is a complex homohexamer made up of three alpha‐helical dimers forming a three‐blade propeller‐like structure (Figure 2).^17^ They found each of the six monomers to associate with two Mg^2+^ ions and one GGPP molecule. However, more recent studies suggested that three Mg^2+^ are required for substrate binding.^18^ Such discrepancies are most likely due to the low resolution at which the enzyme was characterised.^18^, ^19^ Miyagi et al. also reported GGDPS to form an octamer in its active form, proving the need for more information on the quaternary structure of this enzyme.^20^ Interestingly, sequence analysis found that this complex quaternary structure is specific to mammalian and insect GGDPS.^17^ Other plant, fungal, archaeal and bacterial orthologues lack the conserved residues that form the enzyme's inter‐dimer region.^17^ This is because in eukaryotes, GGPP production follows the addition of 5‐carbon IPP to FPP while the plant, fungal, archaeal and bacterial orthologues catalyse the condensation reaction of three IPP substrates to the allyl head of DMAPP.^21^, ^22^

With different quaternary structures and only 17% sequence identity, FDPS and GGDPS have significantly similar tertiary structures.^17^, ^23^ Both enzymes contain five conserved regions (I–V) maintained amongst all trans‐prenyltransferases (Figure 2). Regions II and V are made up of aspartate‐rich motifs (DDXXD/N) involved in ligation of Mg^2+^ ions and association with the pyrophosphate on the allylic substrate. Region III (GQXXD) contains a Gln185 which also facilitates this process by providing a polar contact for the allylic tail and Mg^2+^ ions. Region I (GKXXR) contains basic residues, specifically Arg28, His57, Arg73 and Arg74, responsible for pyrophosphate binding of IPP in the homoallylic subpocket. Region IV (KT) provides a Thr152 and Lys151 that stabilise the carbocation intermediate.

Conservation of these key residues may explain the near‐identical catalytic mechanism of both FDPS and GGDPS. This mechanism is proposed to be a three‐step ionisation–condensation–elimination reaction.^24^ First, the enzyme binds IPP and the allylic substrate in their respective pockets. Then, the allylic carbocation is formed by removal of the IPP tail, facilitated by the three Mg^2+^ ions bound in the catalytic cavity. The C1 carbon on the carbocation electrophilically attacks the C4 carbon on the IPP substrate forming a C—C bond. The final product is formed from stereospecific elimination of a proton.

Variation between these two isozymes arises when comparing the hydrophobic channel for the allylic substrate isoprenyl tail. FDPS contains two capping phenyl residues in this cavity (Phe89/99) which are expected to limit chain length (Figure 2). GGDPS instead contains Ala59 and Ser60 which allows for the larger C20 GGPP product.^17^ Interestingly, GGDPS has a second hydrophobic cavity below its active site. Kavanagh et al. determined this second site to represent an inhibitory binding site upon realisation that the aliphatic GGPP product tail does not extend into the former elongation site, but instead into this second binding cavity. This GGPP molecule was found to bind the aspartate motifs in the allylic site rather than the basic residues in the homoallylic site, suggesting an inhibitory feedback mechanism for product regulation. This was confirmed by crystallography studies showing a GGPP analogue, 3‐azaGGPP, acting as a competitive inhibitor with respect to FPP.^25^

## 4 GGDPS REGULATION

**FIGURE 3:**
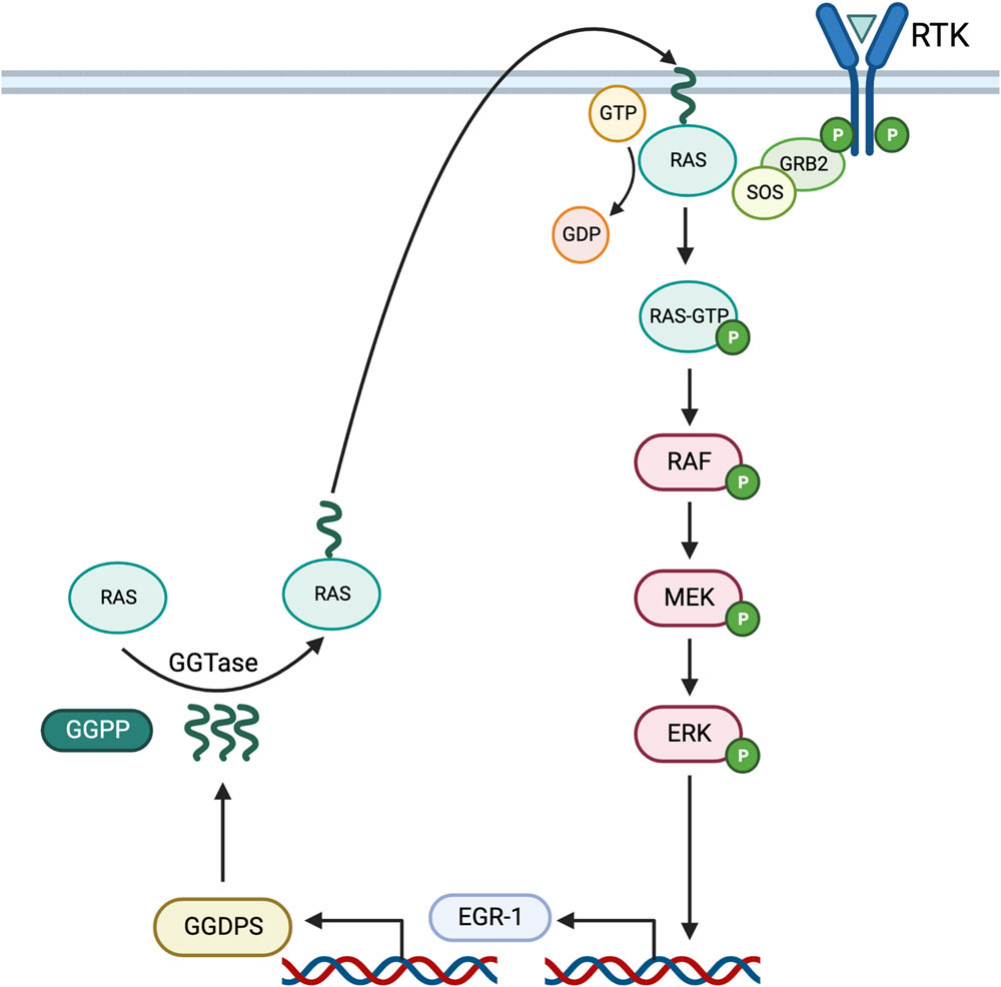
Ras/ERK/EGR1 pathway regulation of GGDPS gene expression. GGDPS has recently been identified as a target gene for EGR1. Activation of the MAPK signalling pathway initiates EGR1 expression and GGDPS expression. GGDPS expression allows for production of GGPP, the substrate utilised by GGTases for geranylgeranylation of target proteins (ex: K‐Ras). Prenylation of K‐Ras promotes Ras membrane localisation allowing for the reactivation of the ERK pathway in the presence of insulin or other pathway activators, creating a positive feedback loop. Note: K‐Ras is usually farnesylated by FTase, however in regard to this pathway promoting GGDPS expression, it was shown to be primarily geranylgeranylated by GGTase I.

Regulation of GGDPS activity primarily stems from product inhibition.^17^, ^26^, ^27^, ^28^ As mentioned earlier, GGDPS has an inhibitory binding site which binds GGPP and renders the enzyme inactive. This creates a negative feedback loop to maintain homeostatic GGPP levels. Recent studies have also implicated regulation of the enzyme at the transcriptional level. Activation of the extracellular signal‐regulated kinase (ERK) pathway initiates translocation of transcription factor early growth response gene 1 (EGR1) which promotes expression of GGDPS as well as expression of other cholesterol biosynthesis enzymes such as HMGCR^29^, ^30^, ^31^, ^32^, ^33^ (Figure 3). Activation of the ERK pathway and subsequent EGR1‐initiated GGDPS expression can occur in response to changes in insulin levels, consistent with the insulin‐dependent induction of cholesterol biosynthesis that is found in the liver. Recent evidence also suggests a role of EGR2 in regulation of GGDPS expression.^34^ Transcriptional regulation of GGDPS is also supported by the finding of two different GGDPS mRNAs in 16 different human tissues, as well as several partial cDNA sequences, suggesting the existence of additional mRNAs.^23^, ^35^ The two identified mRNAs were most abundant in the heart, skeletal muscle and testis and the shorter mRNA was the major species out of the two.^23^ However, the functional consequences of the different mRNAs have not yet been delineated.

Regulation of cholesterol biosynthesis enzymes, specifically HMGCR, result from binding of sterol regulatory binding element (SREBP) transcription factors.^36^, ^37^, ^38^, ^39^ SREBPs operate in a negative feedback loop which, when cholesterol levels are high, leads to the degradation of HMGCR.^40^ A SRE has not been identified in the GGPS1 gene, which encodes GGDPS, suggesting GGDPS expression is not dependent on SREBPs. This is supported by the finding that while sterol accumulation leads to decreased mRNA expression of enzymes such as HMGCR and FDPS, GGDPS mRNA levels are unaffected by changes in cellular sterol levels.^35^

## 5 GGDPS EXPRESSION AND KNOWN MUTATIONS

**FIGURE 4:**
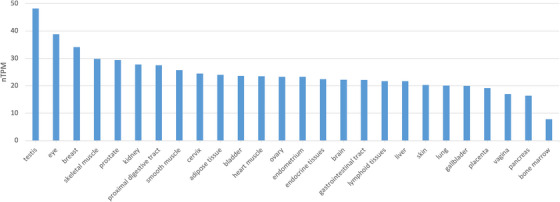
GGPS1 expression across tissues. Expression values are shown in nTPM (normalised transcripts per million) created by combining the HPA and GTEx transcriptomics datasets using the Human Protein Atlas normalisation pipeline. Data were obtained from the Human Protein Atlas (https://www.proteinatlas.org/ENSG00000152904‐GGPS1/tissue).

Due to GGDPS's role in isoprenoid biosynthesis and metabolism, it is ubiquitously expressed in all tissues (Figure 4). Based on data from the Human Protein Atlas, *GGPS1* expression is highest in the testis which may be related to the essential role of GGDPS in spermatogenesis.^35^, ^41^, ^42^ Expression levels also appear elevated in the eye, skeletal muscle and breast tissue, suggesting that GGDPS may play an important role in these tissues as well. While previous studies have found elevated levels of geranylgeranylated proteins in the human heart, brain, skeletal muscle and testis, variation between geranylgeranylated protein levels and *GGPS1* tissue expression may indicate that expression of the synthase does not necessarily correlate with levels of geranylgeranylated proteins.^23^

**FIGURE 5:**
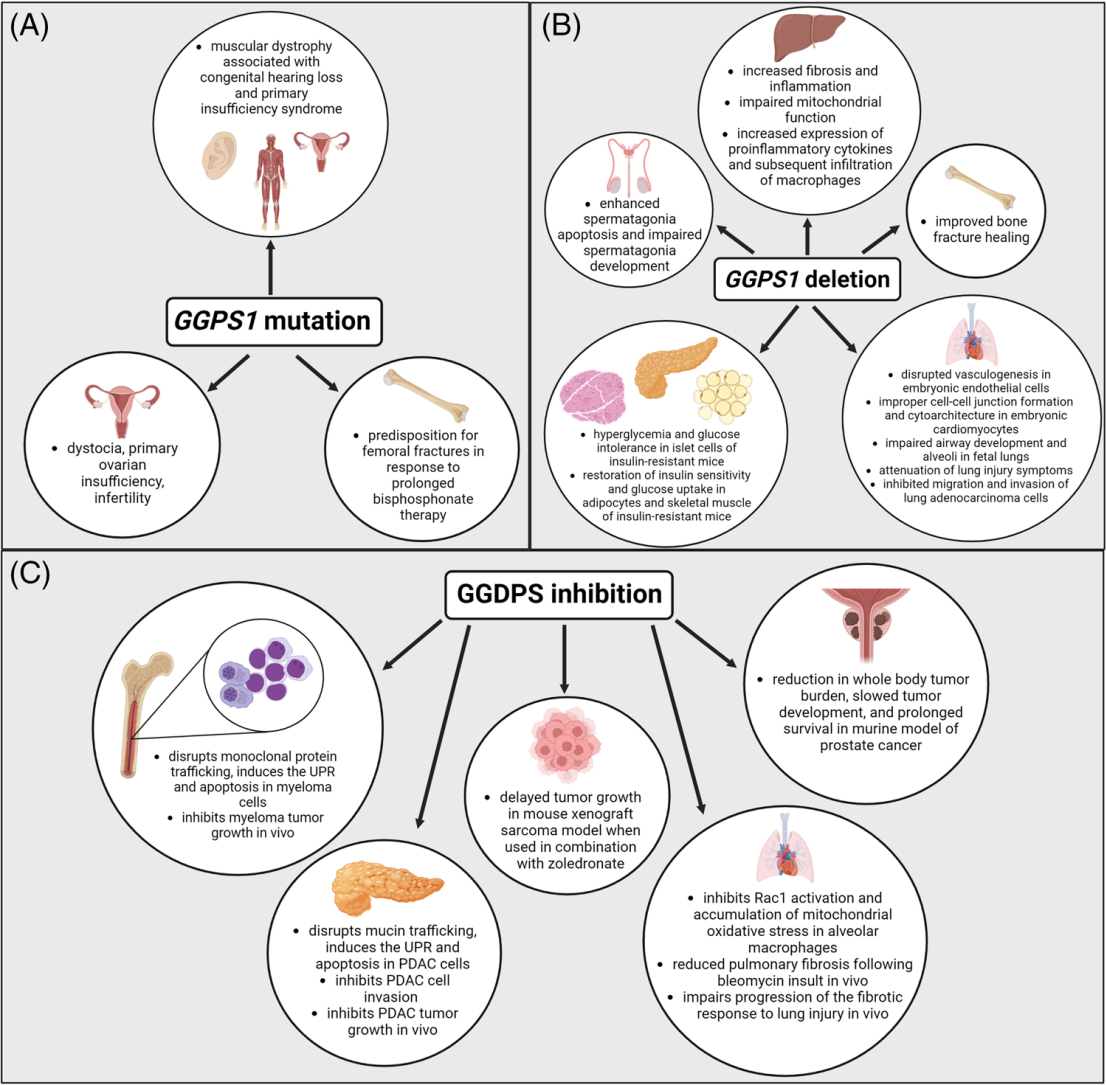
Pathophysiological processes and disease states influenced by GGPS1 mutations, tissue‐specific GGPS1 deletions and GGSIs. (A) GGPS1 mutations have been associated with a unique syndrome of muscular dystrophy associated with congenital hearing loss and primary ovarian insufficiency. GGPS1 SNPs have also been associated with bone disease and theorised to cause dystocia and infertility issues in women. (B) Tissue‐specific KO models for spermatogenesis, cardiovascular and thoracic development, lung injury, insulin‐resistance, liver disease and bone health have uncovered possible roles of GGDPS in disease. (C) GGSIs show potential benefit in models of multiple myeloma, pancreatic ductal adenocarcinoma (PDAC), sarcoma, pulmonary fibrosis and prostate cancer.

Recent studies have shown a mutation in the *GGPS1* gene was associated with a unique case of muscular dystrophy associated with congenital hearing loss and primary ovarian insufficiency (Figure 5A).^43^ Multiple instances of biallelic missense mutations were found to cause this syndrome.^43^, ^44^ Functional assays revealed that the activity of the mutated GGDPS was only moderately impaired (∼50%), which would not be expected to elicit a phenotype. Because of this, the investigators hypothesised that the described phenotype was perhaps a consequence of a more subtle change in GGDPS function, such as affecting subcellular localisation of the enzyme for cell‐type specific processes.^43^ Interestingly, attempts to knock‐in this mutation resulted in embryonic lethality, potentially through underdevelopment of the placental/embryonic vascular unit.^43^ This finding is consistent with the observation that *GGPS1* is essential for folliculogenesis and oocyte maturation, as *GGPS1* deficiency in oocytes impacts female fertility in a stage‐specific manner.^45^ It is theorised that *GGPS1* deficiency or single nucleotide polymorphisms (SNPs) in somatic cells of the uterus may be responsible for dystocia, or difficulty during the delivery process. However, this has not been confirmed since most females with *GGPS1* mutations suffer from primary ovarian insufficiency and infertility.^46^ Decreased *GGPS1* expression in the testis has also been associated with infertility in men, suggesting a role for this enzyme in both male and female reproductive organs.^41^, ^42^, ^47^, ^48^

*GGPS1* SNPs have also been identified in connection with bone remodelling. Multiple studies have found various *GGPS1* mutations to contribute to predisposition of femoral fractures in response to prolonged bisphosphonate therapy.^49^, ^50^, ^51^, ^52^ The mechanism underlying this predisposition is not fully understood, but data suggest the fractures may result from the combination of impaired FDPS activity (secondary to the bisphosphonate) and GGDPS activity (secondary to the mutation).^51^

## 6 KNOCKOUT MODELS OF GGDPS

Various knockout models have elucidated a critical role of GGDPS in embryonic development (Figure 5B). A knock‐in mouse model was generated to study the effect of the Y259C missense mutation known to contribute to muscular dystrophy associated with congenital hearing loss and primary ovarian insufficiency which was mentioned above.^43^ This mutation led to embryonic lethality due to improper embryonic vascularisation and cell–cell junction formation. A similar effect resulted from *GGPS1* deletion in mouse embryonic cardiomyocytes. The enzyme proved to be essential for proper cardiac cytoarchitecture and cell–cell junction formation.^53^ Similarly, knockout of GGDPS in mouse embryonic endothelial cells disrupted vasculogenesis causing embryonic lethality, while deletion of the gene in foetal lungs was shown to impair proper development of airways and alveoli.^54^

The role of GGDPS in fertility has also been explored using knock‐out models. Loss of GGDPS in Sertoli cells was found to enhance spermatogonia apoptosis and block further spermatogonia development.^47^ Deletion in myometrial cells was found to impair uterine contractions resulting in dystocia and disrupted embryonic placing, while deletion in oocytes was associated with ovarian dysfunction and infertility.^44^, ^45^

Connections between GGDPS and non‐alcoholic fatty liver disease (NAFLD) have been identified. Liver‐specific deletion of GGDPS was found to reprogram hepatic metabolism toward glycolysis, resulting in fibrosis and inflammation.^55^ In addition, liver‐specific GGDPS deletion impaired mitochondrial function by disrupting modification of essential mitochondrial proteins such as Rab7, which is important for mitophagy and mitochondrial fission, further contributing to conversion toward a glycolytic phenotype and liver fibrosis.^55^ GGPP also acts as a precursor for coenzyme Q (CoQ) which functions as an electron transporter in the mitochondrial electron transport chain. Therefore, loss of GGDPS and depletion of GGPP may disrupt CoQ synthesis and oxidative phosphorylation.

GGDPS has also been implicated in T2D through an EGR1/GGDPS/Ras/ERK1/2/IRS‐1 pathway‐dependent manner.^31^ Knockout of GGDPS in the pancreatic β‐cells of insulin‐resistant mice resulted in hyperglycaemia and glucose intolerance due to β‐cell dysfunction.^56^ However, deletion of GGDPS in the adipocytes and skeletal muscle of insulin‐resistant mice was found to restore insulin sensitivity and glucose uptake.^31^, ^57^ Thus, it is evident that there is a complex and tissue‐specific relationship between GGDPS and T2D that requires further investigation.

Other GGDPS knockout models have focused on ventilator‐induced lung injury and acute lung injury (ALI). In both mouse models, lung‐specific deletion of *GGPS1* attenuated disease symptoms.^58^, ^59^, ^60^ Deletion of *GGPS1* reduced Rab10 membrane localisation which led to decreased activation of TLR4–NF‐κB signaling.^58^ NF‐ κB is a necessary transcription factor for NLRP3 activation, therefore by inhibiting this pathway, NLRP3 inflammasome transcription was inhibited, attenuating disease‐induced lung inflammation.^58^ Further studies found that inhibition of NLRP3 inflammasome by GGDPS inhibition resulted in promotion of autophagy, allowing for attenuation of sepsis‐induced lung injury.^59^ Similarly, knockdown of the enzyme also inhibited migration and invasion of lung adenocarcinoma cancer cells.^61^

Finally, in a mouse model evaluating GGDPS in the context of bone fractures, it was identified that loss of the enzyme improved the fracture healing process.^62^ This was due to activation of the Bmp2/Smad‐dependent Runx2 pathway. Bmp2 is essential for initiation of the fracture healing process by regulating expression of transcription factor Runx2 which can then initiate expression of genes for terminal differentiation of chondrocytes. Bmp2 initiates the phosphorylation of various Smad proteins such as Smad1/5/8, which initiates terminal differentiation of chrondrocytes, or Smad2/3, which inhibits terminal differentiation. *GGPS1* conditional knockdown mice were found to have diminished TGF‐β signalling within the first 14 days post‐fracture. Lack of GGDPS inhibited the Ras/ERK/EGR1 pathway leading to decreased expression of TGF‐β, allowing for terminal differentiation of chondrocytes and accelerated fracture healing. However, Bmp2 signalling was up‐regulated in *GGPS1* KO mice within the first 7 days post‐fracture leading to increased Smad1/5/8 phosphorylation and Runx2 expression. In addition, it was theorised that lack of TGF‐β signalling reduced Smad2/3 expression allowing for decreased inhibition of Runx2. The investigators also reported a significant increase in vascular endothelial growth factor A (Vegfa) expression at 7‐ and 21‐days post‐fracture, indicating that deletion of *GGPS1* increased vasculogenesis further accelerating the fracture healing process.

## 7 GGDPS IN DISEASE STATES

Overexpression of GGDPS has been associated with various clinical pathologies. A positive correlation was found between neurofibrillary tangle (NFT) density, p‐Tau levels and mRNA prevalence of both GGDPS and FDPS in the brains of Alzheimer's disease (AD) patients.^63^ With elevated levels of both FPP and GGPP having been identified in the brains of patients with AD, it has been postulated that elevated GGDPS expression may contribute to NFT and β‐amyloid plaque formation.^64^, ^65^, ^66^

**FIGURE 6:**
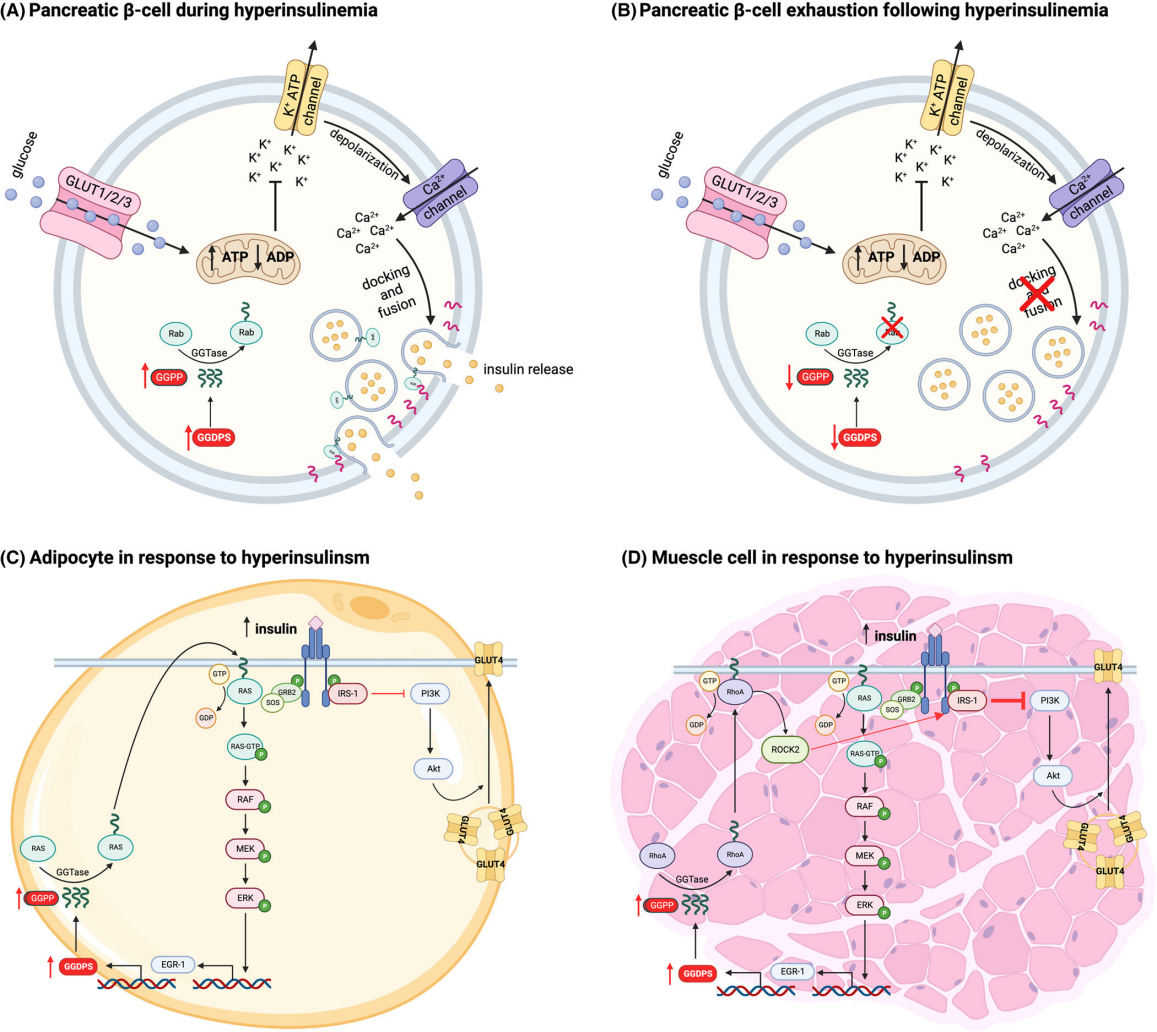
The putative roles of GGDPS in T2D. (A) GGDPS expression is elevated in pancreatic β‐cells during chronic hyperglycaemic challenge. Increased blood glucose stimulates release of the insulin granule pool. Docking and fusion of insulin granules is facilitated by Rab proteins which require geranylgeranylation in order to localise and function properly. (B) Resulting hyperinsulinemia and increased secretory demand may be the cause of pancreatic β‐cell exhaustion accompanied by decreased expression of GGDPS. Decreased enzyme expression diminishes intracellular GGPP levels, inhibiting geranylgeranylation of Rab proteins and subsequently disrupting the docking and fusion of insulin granules. (C) GGDPS is up‐regulated in adipocytes during hyperinsulinemia due to continuous activation of the Ras/ERK/EGR1 pathway. Continuous activation of the pathway leads to phosphorylation of IRS‐1 at the inhibitory serine position, disrupting the PI3K/Akt pathway and translocation of GLUT4 to the membrane. (D) GGDPS is up‐regulated in skeletal muscle during hyperinsulinemia due to continuous activation of the Ras/ERK/EGR1 pathway. In the skeletal muscle, RhoA is continuously geranylgeranylated allowing for activation of ROCK2. Activation of the RhoA/ROCK pathway contributes to the inhibitory phosphorylation of IRS‐1 disrupting GLUT4 translocation.

As previously mentioned, aberrant levels of GGDPS have also been implicated in T2D (Figure 6). Interestingly, GGDPS is overexpressed in the liver, skeletal muscle and adipose tissue of mice with obesity, insulin resistance and hyperinsulinemia.^57^, ^67^, ^68^ However, GGDPS expression was noted to be significantly decreased in the islet cells of T2D patients in response to β‐cell dysfunction.^56^ In normoglycaemic patients, glucose challenge is met with a biphasic response in which the initial insulin release phase relies on the docked insulin granule pool to facilitate glucose uptake and glycogen synthesis.^69^ Patients with T2D have been shown to have a decrease in insulin granules docked at the plasma membrane, diminishing glucose‐responsive insulin release.^56^

GGDPS has been shown to be up‐regulated in β‐cells during the compensatory hyperinsulinemia period in db/db mice.^57^ However, GGDPS was found to be down‐regulated following prolonged excessive insulin release and subsequent β‐cell exhaustion.^56^ It is theorised that such β‐cell exhaustion is due to increased secretory demand in response to chronic hyperglycaemic challenge.^69^ This theory is supported by evidence that GGDPS deficiency contributes to depletion of the docked granule pool due to decreased geranylgeranylation of Rab27a which is required for insulin granule docking.^56^ The reason for decreased expression of GGDPS during β‐cell exhaustion is not fully understood, but may be due to oxidative stress, ER stress or the result of prolonged β‐cell use.^70^

Notably, geranylgeranylation of proteins such as Rab27a, Rab3, Cdc42 and RhoA are essential for proper insulin secretion. During β‐cell exhaustion when GGDPS expression is decreased, expression of these small GTPases is also decreased, thereby disrupting insulin trafficking and secretion.^56^, ^67^ Decreased levels of GGDPS activity may also impact glucose homeostasis by disrupting proper localisation of Rac1. Rac1 is important in the vesicular trafficking of GLUT4 glucose transporters in skeletal muscle and fat cells and disruption of GLUT4 transport to the membrane results in loss of glucose‐stimulated insulin secretion.^31^, ^67^, ^68^, ^71^

EGR1 was found to be highly expressed in the adipose tissue of T2D patients, thus providing a mechanism underlying the increased expression of GGDPS.^31^ EGR1 responds to insulin stimulation, therefore GGDPS expression is sustained in response to hyperinsulinism eventually leading to insulin resistance.^31^ Mechanistically, EGR1 increases in response to hyperinsulinism subsequently activating GGDPS transcription. GGDPS expression then promotes K‐Ras membrane association allowing for the reactivation of the ERK1/2 pathway in the presence of insulin. K‐Ras is usually a substrate of farnesylation but studies have shown it can also be geranylgeranylated by GGTase I.^8^ Through the use of FTase and GGTase I inhibitors respectively, it was determined that it was the geranylgeranylation of K‐Ras that was necessary for the continued activation of the pathway.^30^ Sustained activation of this pathway during hyperinsulinemia has also been shown to cause desensitisation of the PI3K/Akt pathway.^32^ Sustained activation results in phosphorylation of insulin receptor substrate‐1 (IRS1) at the inhibitory serine position.^31^ This disrupts PI3K/Akt pathway activation impairing translocation of GLUT4 transporters and exacerbating insulin resistance.^31^ A similar mechanism was found in the skeletal muscle of obese and insulin resistant mice albeit through activation of the RhoA/ROCK pathway due to GGDPS‐mediated geranylgeranylation.^57^

**FIGURE 7:**
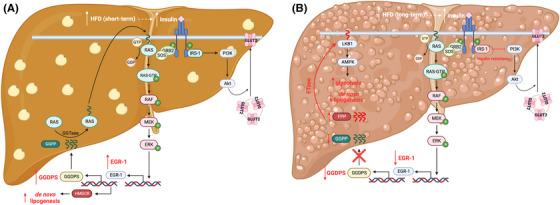
The putative roles of GGDPS in liver disease. (A) Short‐term high‐fat diet (HFD) leads to increased insulin levels and continuous activation of the Ras/ERK/EGR1 pathway. EGR1 induces the expression of GGDPS and HMGCR promoting continuous activation of the Ras/ERK/EGR1 pathway and de novo lipogenesis causing gradual fat accumulation and development of NAFLD. (B) Long‐term HFD leads to decreased EGR1 and GGDPS expression. This inhibits the production of GGPP resulting in the accumulation of FPP. FPP is then utilised by FTase for the farnesylation of LKB1 activating the LKB1/AMPK pathway causing a metabolic shift towards glycolysis. This metabolic shift leads to hepatic inflammation and fibrosis. Continuous activation of the Ras/ERK/EGR1 pathway from HFD also leads to insulin resistance which is characteristic of NASH indicating liver disease progression towards HCC.

Progression of NAFLD to hepatocellular carcinoma (HCC) has been associated with aberrant GGDPS expression.^38^ Studies have found GGDPS down‐regulation to be a possible predictive factor for progression of NAFLD to fibrosis, and lower expression of the enzyme also has been shown to predict recurrence of HCC.^55^ Similar to T2D, the mechanism behind GGDPS expression and NAFLD progression seems to be related to the Ras/ERK/EGR1 pathway (Figure 7). Short‐term exposure to a high‐fat diet (HFD) has been shown to increase EGR1 expression and GGDPS expression. Expression of EGR1 also initiates expression of HMGCR leading to de novo lipogenesis and subsequent fat accumulation. Continuous activation of this pathway due to long‐term exposure to HFD leads to decreased insulin sensitivity and down‐regulation of EGR1 and GGDPS. Without sufficient production of GGPP, FPP accumulates leading to the farnesylation of liver kinase B1 (LKB1). LKB1 activates the AMP‐activated protein kinase (AMPK) pathway leading to mitochondrial dysfunction and metabolic reprogramming to a glycolytic phenotype.^55^ This results in hepatic inflammation through increased release of pro‐inflammatory cytokines and macrophage infiltration, leading to hepatic fibrosis. This theorised mechanism is supported by biopsies from patients with NAFLD without fibrosis and inflammation which showed increased GGDPS expression whereas decreased expression of the enzyme was found in more advanced NAFLD cases.^55^ Non‐alcoholic steatohepatitis (NASH) proceeds NAFLD in the progression of HCC and initial characteristics of NASH include insulin resistance resulting from elevated EGR1 expression in response to long‐term HFD.^55^ Along with the theory of EGR1‐induced NAFLD progression, advanced stages of NASH show decreased EGR1 expression due to chronic insulin insult. Therefore, the down‐regulation of GGDPS is also associated with the advancement of NASH leading to HCC. Interestingly, with respect to virus‐related HCC, GGPS1 mRNA and protein expression levels were found to be up‐regulated in HCC tumour tissue compared with adjacent non‐malignant tissue.^72^ Additionally elevated mRNA and protein expression levels were associated with pathological indicators of advanced disease stage further revealing the complicated and specific role of GGDPS homeostasis in liver disease.

GGDPS has been implicated in inflammatory responses of ALI and acute respiratory distress syndrome (ARDS). The enzyme was found to have significantly increased expression in the alveolar macrophages isolated from patients with ARDS and ALI‐induced mice.^73^ It was also shown that cigarette smoke extract induces GGDPS expression in an EGR1‐dependent manner resulting in constitutive activation of the Ras/ERK/EGR1 pathway.^30^ GGDPS expression was increased in the lung tissues of bleomycin‐induced lung injury mice following lung injury and fibrosis. Enzyme deficiency was found to augment lung fibrosis suggesting a protective role of GGDPS in pulmonary fibrosis.^74^, ^75^ GGDPS expression was significantly increased in lung adenocarcinoma tissues compared with adjacent normal tissues, and expression level was found to correlate with indicators of disease stage such as large tumours, high TNM stage, lymph node metastasis and poor prognosis.^61^ Similarly, GGDPS expression was found to be negatively associated with survival in patients with small cell lung cancer.^76^

## 8 IBP INHIBITORS

Altered metabolism is a hallmark of cancer, making the IBP a target of interest. The IBP is essential for both sterol and non‐sterol synthesis and has also been associated with cell survival and proliferation.^77^, ^78^ The cellular effects due to IBP inhibition vary depending on the specific enzyme that is targeted. The following classes of IBP inhibitors that have proven useful in understanding the effects of depleting intracellular GGPP levels and globally disrupting protein geranylgeranylation include statins, nitrogenous bisphosphonates (NBPs) and GGDPS inhibitors (GGSIs). Statin‐mediated inhibition of HMGCR disrupts cholesterol synthesis, but also disrupts synthesis of isoprenoid intermediates (FPP and GGPP). Likewise, the NBPs, by virtue of inhibiting FDPS, disrupt not only sterol synthesis but also GGPP synthesis. Finally, direct inhibitors of GGDPS do not impact sterol synthesis, but do impact synthesis of compounds distal to GGPP synthesis in the pathway. While all three classes of drugs have proven useful in elucidating the effects of disrupting isoprenoid synthesis and protein prenylation in vitro, only the GGSIs have the potential to directly address disease pathologies characterised by aberrant GGDPS activity and/or dependence on GGPP production.

## 9 STATINS

Statins are the most widely clinically used IBP inhibitors. These agents inhibit the rate‐limiting enzyme HMGCR, thereby impacting both sterol and non‐sterol synthesis. Clinically they are used for the management of hypercholesterolemia. Reduction in sterol synthesis induces the cleavage and translocation of SREBPs, subsequently regulating synthesis of cholesterol biosynthesis enzymes as well as increasing expression of LDL receptors.^36^, ^37^ In addition to lowering LDL‐cholesterol levels, statins have also been shown to inhibit neovascularisation and regulate superoxide levels protecting against a wide variety of cardiovascular diseases, including atherosclerosis, ischemia–reperfusion injury, arrhythmia, cardiac fibrosis and pulmonary hypertension.^79^, ^80^, ^81^, ^82^, ^83^, ^84^, ^85^, ^86^, ^87^, ^88^, ^89^

While statins are most commonly used for management of hypercholesteremia, there has been significant interest in their potential as anti‐cancer agents.^90^ In vitro studies in various malignancies such as breast, ovarian, colon, prostate, lung adenocarcinoma, mesothelioma, acute myeloid leukaemia and multiple myeloma have shown that statins inhibit cell proliferation and invasion and induce apoptosis.^61^, ^91^, ^92^, ^93^, ^94^, ^95^, ^96^, ^97^, ^98^, ^99^, ^100^, ^101^, ^102^, ^103^, ^104^, ^105^, ^106^, ^107^, ^108^, ^109^, ^110^ Such statin‐induced apoptosis has been confirmed to be the result of GGPP depletion rather than other products downstream of HMGCR such as mevalonate or FPP.^111^, ^112^ Further evidence supporting the importance of GGPP depletion in the anti‐cancer properties of statins is the finding that disruption of RhoA geranylgeranylation impairs metastasis due to disruption of cell attachment, invasion and migration.^92^, ^111^

However, while these in vitro findings are intriguing, it is noted that the concentrations required to disrupt protein prenylation (typically > 1 μM) are much higher than those required to inhibit cholesterol biosynthesis,^26^ thus standard doses of clinically utilised statins are unlikely to impact protein prenylation in vivo.^113^, ^114^, ^115^, ^116^, ^117^ While several phase 1 trials showed higher doses of statins could result in serum levels in the micromolar range, these doses were also associated with unwanted side effects with minimal anti‐tumour effects.^114^, ^118^ In the Thibault et al., phase I study, it was noted that myopathy was the dose‐limiting toxicity of high‐dose lovastatin treatment and that treatment with ubiquinone could both reverse and prevent this toxicity.^118^ Myopathy was not noted in another phase I study of high‐dose lovastatin, perhaps secondary to differences in treatment duration.^119^

A few prospective randomised trials have evaluated whether the addition of statins to standard chemotherapy agents/regimens improve anti‐tumour efficacy or survival outcomes (Table 1).^120^, ^121^, ^122^, ^123^, ^124^, ^125^, ^126^, ^127^ In aggregate these studies have shown that standard doses of statins failed to significantly improve response rates or survival outcomes in a variety of solid tumour populations. A study in which patients received adjuvant pravastatin following completion of transarterial embolisation and 5‐fluorouracil treatment for advanced HCC, reported improvement in median overall survival (18 months for the pravastatin group vs. 9 months for the observation group, *p* = .006).^122^ Overall, the generally negative outcomes of these randomised studies are most likely a consequence of standard statin dosing being insufficient to significantly impair protein geranylgeranylation in vivo.

**TABLE 1:** Summary of randomised trials involving statin/chemotherapy combinations in advanced malignancies

Reference	Disease	Trial phase	Treatment	No. of patients	Primary outcome (statin vs. placebo/observation)
Yulian et al.^120^	Locally advanced breast cancer	II	FAC + simvastatin (40 mg)/placebo	66	ORR: 90 vs. 70%, *p* = .103
Han et al.^121^	Relapsed/refractory advanced non‐small cell lung cancer	II	Gefitinib ± simvastatin (40 mg)	106	ORR: 38.5 vs. 31.5%, *p* = .666
Konings et al.^125^	Advanced gastric cancer	II	ECC ± pravastatin (40 mg/d)	30	6‐mos PFS: 43 vs. 47%
Hong et al.^127^	Advanced pancreatic cancer	II	Gemcitabine + simvastatin (40 mg)/placebo	114	TTP: 2.4 vs. 3.6 mos; *p* = .903
Seckl et al.^123^	Newly diagnosed small cell lung cancer	III	Cisplatin/etoposide + pravastatin (40 mg)/placebo	846	OS: median 10.7 vs. 10.6 mos, *p* = .9
Lim et al.^124^	Relapsed/refractory metastatic colorectal cancer	III	FOLFIRI/XELIRI + simvastatin (40 mg)/placebo	269	PFS: median 5.9 vs. 7.0 mos, *p* = .937
Kim et al.^126^	Advanced gastric cancer	III	XP + simvastatin (40 mg)/placebo	244	PFS: median 5.2 vs. 4.6 mos, *p* = .642

Abbreviations: ECC, epirubicin/cisplatin/capecitabine; FAC, fluorouracil/doxorubicin/cyclophosphamide; FOLFIRI, fluorouracil/leucovorin/irinotecan; ORR, overall response rate; OS, overall survival; PFS, progression free survival; TAE, transarterial embolisation; TTP, time to progression; XELIRI, capecitabine/irinotecan; XP, capecitabine/cisplatin.

## 10 NITROGENOUS BISPHOSPHONATES

Bisphosphonate‐based drugs have been around for over 50 years.^128^ Bisphosphonates act as non‐hydrolysable analogues of inorganic pyrophosphate that share a common phosphorous–carbon–phosphorous backbone which coordinates the chelation of calcium ions. Originally these drugs were determined useful because of their calcium binding properties, rendering them a treatment for bone disease. More recently developed bisphosphonates, such as zoledronic acid, were discovered to inhibit FDPS and osteoclast resorption. These inhibitors belong to the class of NBPs which are approved for treatment of various bone diseases such as osteoporosis, metastatic bone disease and myeloma bone disease. While these agents specifically inhibit FDPS, it was discovered that the mechanism behind their effects on osteoclasts was due to downstream depletion of GGPP.^129^, ^130^, ^195^

In vitro studies have shown NBPs to have a number of anti‐tumour effects, including inhibiting tumour cell proliferation, inducing apoptosis, inhibiting adhesion and invasion, having anti‐angiogenic properties as well as synergistic effects with standard anti‐neoplastic drugs.^132^, ^133^, ^134^, ^135^, ^136^, ^137^, ^138^, ^139^, ^140^, ^141^, ^142^, ^143^ In several studies, depletion of GGPP was noted to be a key mechanism underlying the observed anti‐tumour effects.^144^, ^145^, ^146^, ^147^ There have also been several studies that demonstrated that NBPs have immunomodulatory activities as a result of activation and proliferation of Vγ9Vδ2 T cells, leading to anti‐cancer activity.^136^, ^148^, ^149^, ^150^ The underlying mechanism for this phenomenon is related to NBP‐induced increase in intracellular IPP levels, as both IPP and ApppI (an ATP analogue resulting from covalent binding of IPP to AMP) serve as phosphoantigens which stimulate Vγ9Vδ2 T cell expansion.^151^, ^152^, ^153^, ^154^

In clinical studies, NBPs have been shown to decrease skeletal morbidity in multiple myeloma, breast cancer, prostate cancer, lung cancer and other tumours that metastasize to the bone.^155^, ^156^, ^157^, ^158^ There has been less certainty as to whether NBPs impact survival outcomes in a manner independent of the effects on skeletal‐related events (SREs). In a phase III clinical trial evaluating the effects of bisphosphonate and thalidomide therapy for newly diagnosed multiple myeloma patients, zoledronic acid was found to improve overall survival and progression‐free survival while significantly lowering the SRE risk.^159^, ^160^ The effect of zoledronic acid on overall survival was reported to be independent of the reduction in SREs, suggesting more direct anti‐myeloma effects,^159^, ^160^ although a subsequent analysis of the data suggested this effect did not quite reach statistical significance (*p* = .0515).^161^ A meta‐analysis of eight randomised studies involving patients with early stage breast cancer suggested that adjuvant bisphosphonate use was associated with a reduction in the rate of breast cancer recurrence in the bone (relative risk 0.83, *p* = .004) and modest reduction in breast cancer mortality (relative risk 0.91, *p* = .04).^162^ Several studies have suggested more direct anti‐tumour activity of zoledronic acid in the setting of breast cancer where enhanced tumour cell apoptosis or clearance of bone marrow disseminated tumour cells have been observed.^163^, ^164^, ^165^ However, there are also multiple randomised trials that have failed to demonstrate improvement in non‐bone related outcomes.^166^, ^167^, ^168^, ^169^, ^170^, ^171^, ^172^, ^173^ Overall, it has not been apparent that NBPs have substantial anti‐cancer activities outside of the setting of bone marrow/bone disease, which is likely a consequence of their limited systemic distribution due to their high bone affinity.^174^, ^175^

## 11 GGDPS INHIBITORS

Given the therapeutic interest in more selectively targeting GGDPS (and protein geranylgeranylation) without impacting processes upstream in the IBP, efforts towards developing specific GGSIs were initiated. Early generations of GGSIs included bisphosphonates containing isoprenoid substituents.^176^ One of the first discovered GGSIs, digeranyl bisphosphonate (DGBP), was found to inhibit GGDPS with an IC_50_ of around 200 nM.^177^ While crystallography studies evaluating DGBP binding to human GGDPS have not been reported, studies in *Saccharomyces cerevisae* revealed that at least one geranyl chain on the central carbon of the bisphosphonate headgroup is required for specific inhibition of GGDPS. The bisphosphonate head group was found to complex with the magnesium ions with the two prenyl side chains occupying the FPP binding site and the GGPP product‐binding site.^178^

Later advancements led to the development of isoprenoid bisphosphonates containing a triazole linker group.^179^ Extensive structure‐function analysis of a series of triazole bisphosphonates revealed the importance of alkyl chain length and olefin stereochemistry in determining inhibitor potency, and later studies showed the impact of different substituents at the α‐position.^179^, ^180^, ^181^, ^182^, ^183^, ^184^

Other bisphosphonate‐based GGSIs include thienopyrimidine bisphosphonate‐based (Th‐BP) compounds. The extension of the thienopyrimidine moiety was shown to increase potency due to its extension into the IPP binding site. However, this was only seen in compounds with a modification at the C‐2 position, while modification at the C‐6 position was found to increase potency for inhibition of FDPS instead.^185^, ^186^

Studies are ongoing to understand the systemic effects of GGSIs. In studies involving triazole bisphosphonate GGSIs, hepatoxicity was determined to be the dose‐limiting toxicity, with no effects observed on haematological, cardiac or renal parameters.^187^, ^188^, ^189^ These studies suggested that the hepatoxicity was a consequence of GGDPS inhibition and not due to the triazole moiety, since the use of a structurally‐similar compound (differing by having one less carbon in the isoprenoid side chain) with ∼400‐fold less potency as a GGSI (RAM3059) did not induce liver damage at doses >threefold higher than the maximal tolerated dose (MTD) of the lead compound.^189^ However, further studies are necessary to confirm that these effects are target based and not specific to the compound structure. It was also confirmed that doses below the MTD could be safely administered and result in inhibition of protein geranylgeranylation in vivo.^189^ In addition, the combination of low‐dose statin with low‐dose GGSI resulted in undetectable hepatic GGPP levels and enhanced the hepatotoxic effects, suggesting an association between on‐target effects (reduction of GGPP) and hepatotoxicity.^188^

A non‐triazole containing GGSI was found to cause significant weight loss in a mouse model of prostate cancer.^190^ The weight loss suggested possible toxicity from the GGSI, however this same result was not seen in non‐tumour bearing mice when treated with the inhibitor, suggesting that the GGSI alone was not responsible for the toxicity.^190^

In 2018, Lacbay et al. screened over 200 Th‐BP analogues and determined one compound (11c) to disrupt protein geranylgeranylation and decrease serum M‐protein levels in a mouse model of myeloma.^185^ Interestingly, following a 10‐day treatment course (3 mg/kg/day via intraperitoneal injection), serological analyses revealed that three out of the seven treated animals had markedly elevated (>10‐fold upper limit of normal (ULN)) AST levels and three more with >twofold ULN increases. Elevations in ALT levels were also observed in five animals, ranging from twofold to 12‐fold ULN. In 2022, Lee et al. conducted further studies with the same Th‐BP GGSI.^186^ Here, they reported that seven days after administration of a single dose of GGSI (up to 10 mg/kg via IV administration), no abnormalities in liver function tests were observed. Whether this dose level was sufficient to substantially alter hepatic GGPP levels or disrupt hepatic protein geranylgeranylation was not disclosed. In addition, the pharmacokinetic studies revealed a terminal half‐life of 5.34 h, significantly shorter than the previously reported triazole bisphosphonates,^187^, ^189^ thus making it difficult to draw any definitive conclusions regarding class effect versus specific agent effect and hepatoxicity.

Finally, a dual FDPS–GGDPS inhibitor also showed potent activity without signs of toxicity in a murine xenograft model utilising SK‐ES‐1 sarcoma cells, although the only reported metric of toxicity was animal weight.^191^ In aggregate, the available preclinical literature has demonstrated the feasibility of systemically administering GGSI therapy, although further studies are needed to better understand the observed hepatic toxicity and thus maximise the therapeutic window.

## 12 THERAPEUTIC EFFECTS OF GGDPS INHIBITORS

In contrast to statins and FDPS inhibitors which globally disrupt sterol synthesis and protein prenylation, inhibition of GGDPS depletes GGPP levels, thereby impacting protein geranylgeranylation, without inhibiting farnesylation or sterol synthesis. It is important to note that both in vitro and in vivo data suggest that the effects of GGDPS inhibition on a myriad of cellular processes are primarily due to disruption of geranylgeranylation.^77^, ^130^, ^131^, ^178^, ^192^, ^193^, ^194^, ^195^ However, there are also data that suggest that GGSI‐induced FPP accumulation can induce apoptosis as a consequence of conversion of FPP to farnesol.^196^

GGDPS inhibition is associated with anti‐proliferative effects in a variety of malignancies as a result of disruption of geranylgeranylation of proteins essential for cell growth and survival processes (Figure 5).^78^, ^146^, ^197^ While Ras proteins are primarily farnesylated by FTase I, the Rho and Rab families are geranylgeranylated by GGTase I and GGTase II respectively. Rho proteins such as Rho, Rac and Cdc42 are important for cytoskeletal reorganisation and regulate dynamics such as cell polarity, cell motility and membrane protrusion. Rab proteins are important in intracellular membrane trafficking and regulate vesicle formation, transport, docking and fusion. Understanding the impact of GGDPS inhibition on Rho and Rab protein localisation and function is essential for understanding the potential therapeutic benefit of these inhibitors.

In vitro studies showed that the GGSI DGBP inhibited cell migration in a human breast cancer cell line MDA‐MB‐231 and induced autophagy in both MDA‐MB‐13 and PC3 prostate cancer cells.^197^, ^198^ LC3‐II accumulation (a marker of autophagic flux) was also evident when multiple myeloma cell lines were treated with DGBP.^199^ However, the use of GGTase I and GGTase II specific inhibitors did not recapitulate the effects of DGBP, indicating that DGBP effects are not solely due to disruption of geranylgeranylation. Another interesting study found that GGSI‐induced GGPP depletion inhibited micropinocytosis of GGPP leading to amino acid starvation and apoptosis.^77^ However, these effects were specific to MCF10A PTEN knockout cells and K‐RasG12V expressing cells, suggesting these effects may be specific to certain oncogenic cell types.

One study found DGBP to inhibit proliferation and induce apoptosis in lymphocytic leukaemia cells more potently than the FDPS inhibitor zoledronate.^146^ Addition of GGPP following DGBP treatment was shown to abrogate the anti‐proliferative effects, indicating GGPP depletion to be the key mechanism underlying DGBP effects. DGBP was also found to induce apoptosis in T‐cell acute lymphoblastic leukaemia (T‐ALL) cell lines.^200^ A potential mechanism was postulated, connecting disruption of Rab7 localisation due to GGPP depletion with inhibition of Notch1 expression.^201^ Notch1 is altered in over 60% of T‐ALL cases and has been shown to promote proliferation and differentiation.^201^ It was concluded that disruption of Rab7 localisation inhibited Notch1 proliferation, allowing for caspase activation and apoptosis of T‐ALL cells.

The GGSI disodium [(6Z,11E,15E)‐9‐[bis(sodiooxy)phosphoryl]‐17‐hydroxy‐2,6,12,6‐tetramethyheptadeca‐2,6,11,15‐tetraen‐9‐yl]phosphonate (GGOHBP) was found to significantly decrease adrenal gland metastasis in a murine model of human prostate cancer.^190^ In a separate study it was also reported to significantly reduce whole body tumour burden, slow tumour development and prolong survival in a murine model of human prostate cancer.^202^ Lipophilic bisphosphonate BPH‐1222 in combination with rapamycin was found to potently suppress tumour growth in a murine model of lung adenocarcinoma.^203^ BPH‐1222 is an analogue of zoledronate that targets both FDPS and GGDPS. Treatment with BPH‐1222 alone was found to block K‐Ras prenylation initiating ER stress and autophagy, but it did not ultimately induce apoptosis. Combination treatment of the bisphosphonate along with rapamycin allowed for the induction of autophagy and concomitant inhibition of the mTOR pathway which had previously allowed for tumour cell survival.^203^ Similarly, a dual FDPS–GGDPS inhibitor was found to delay tumour growth in a mouse xenograft sarcoma model.^191^

In malignancies characterised by aberrant protein secretion, disruption of Rab geranylgeranylation was found to be the primary mechanism underlying GGSI‐induced cytotoxicity. Treatment of multiple myeloma cells with GGSIs or GGTase II inhibitors results in activation of the unfolded protein response (UPR) pathway and apoptosis.^187^, ^192^ Accumulation of monoclonal protein in the ER was shown to cause ER stress and activation of the UPR.^192^ Prolonged activation of the UPR, such as with GGSI‐mediated inhibition of Rab activity, leads to activation of the apoptotic pathway. The previously mentioned novel triazole‐based GGSIs were shown to potently disrupt monoclonal protein secretion in multiple myeloma cells leading to apoptosis.^179^, ^181^ These same triazole bisphosphonates were also found to decrease tumour growth in mice with multiple myeloma flank xenografts.^187^ Similar results were reported with the use of a Th‐BP inhibitor in both in vitro and in vivo models of multiple myeloma.^193^

GGSIs have also shown efficacy in models of pancreatic ductal adenocarcinoma (PDAC). PDAC is characterised by the abnormal production of aberrantly glycosylated mucins.^204^ Treatment of PDAC cell lines with a GGSI was found to disrupt intracellular trafficking of key mucins like MUC1 leading to activation of the UPR and subsequent apoptosis.^194^ These results were recapitulated in vivo where triazole bisphosphonate GGSI therapy significantly slowed tumour growth in two different mouse models of PDAC.^194^

GGSIs may also be therapeutically relevant in bone disorders. Bone diseases are commonly treated with NBPs which result in disruption of cytoskeletal arrangement through disruption of the F‐actin ring and ruffled boarder necessary for osteoclast function.^130^, ^131^, ^195^ As these effects on osteoclast formation result from depletion of GGPP rather than FPP, GGSIs may be an alternative to FDPS inhibitors.^195^ Therefore, the use of GGSIs, especially in the case of bone pain or fractures resulting from the primary disease, such as in the case of multiple myeloma, may hold promise in treating both the primary disease as well as preventing bone resorption. Further potential benefits include the greater systemic distribution of the triazole bisphosphonate GGSIs compared with NBPs.^187^

Finally, there has also been interest in the therapeutic potential of GGSIs in pulmonary fibrosis. DGBP was found to abrogate pulmonary fibrosis in a mouse model utilising bleomycin‐induced lung injury.^74^ DGBP disrupted localisation of Rac1 to the mitochondria of alveolar macrophages, which in turn attenuated mitochondrial oxidative stress levels and limited the fibrotic lung response.^74^

## 13 CONCLUSIONS

In this review, we provide a comprehensive overview of the role of GGDPS in normal physiological processes. While the factors that impact the expression and regulation of the enzyme in pathophysiological conditions remain incompletely understood, it is evident that the EGR1/GGDPS/Ras/ERK1/2 pathway plays an important role. However, whether there are other modulators or pathways that impact GGDPS activity remain to be determined.

**FIGURE 8:**
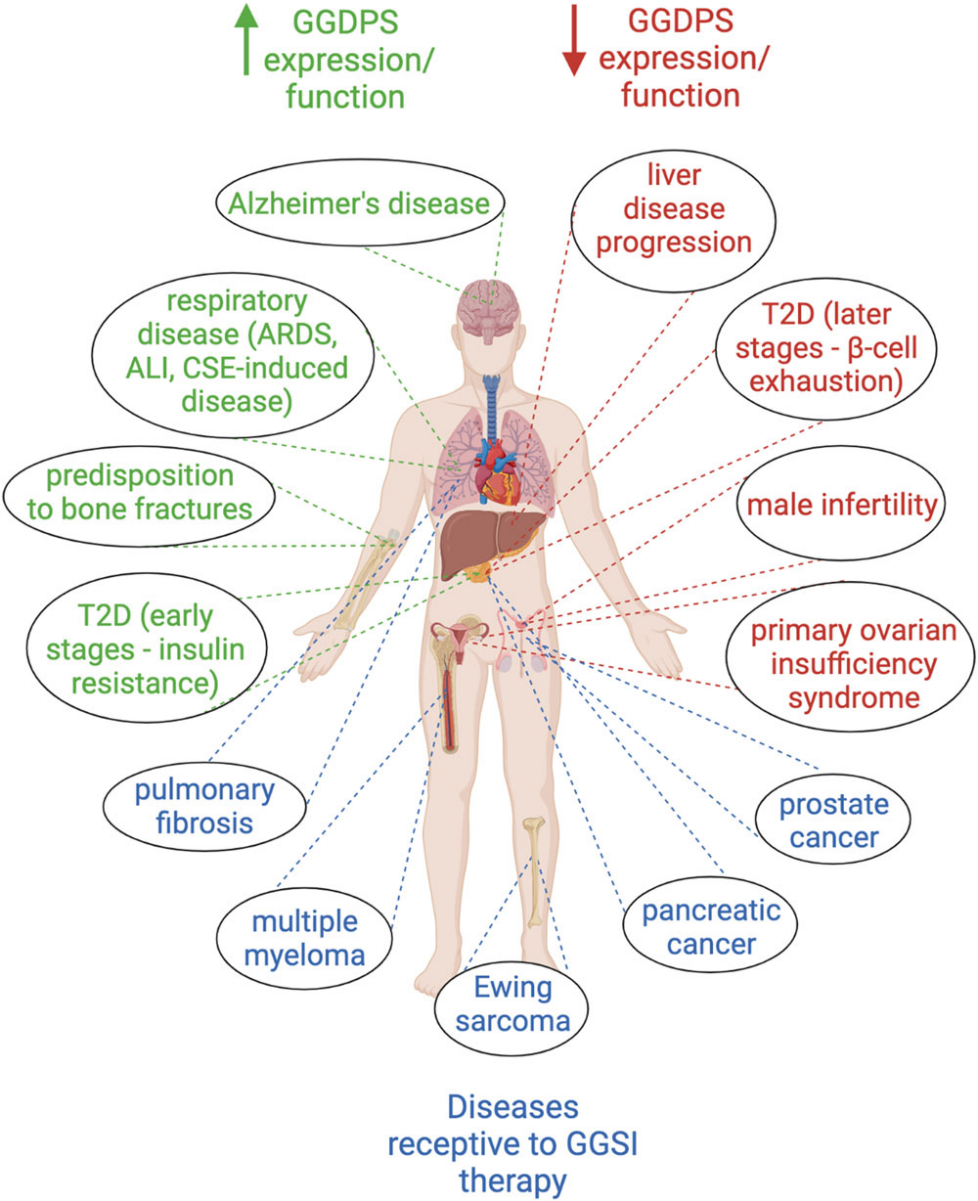
Overview of the pathological processes impacted by altered GGDPS expression or activity

It is evident that GGDPS is a contributor to a wide range of human pathologies, including liver disease, T2D, pulmonary disease and malignancy, and therefore is an enzyme of significant clinical interest (Figure 8). Available preclinical studies involving GGSIs have demonstrated efficacy in several malignancy types that are currently considered incurable (e.g., multiple myeloma and PDAC) (Figure 8). In addition, preclinical studies with a GGSI have revealed efficacy in pulmonary fibrosis, a disease with limited therapeutic options. Overall, there is substantial rationale for the further development of GGSIs and to expand the scope of GGSI therapeutic intervention research to not only include other malignancies, but also other disease states such as liver disease and T2D. Ultimately, successful translation to the clinic will depend not only on understanding the GGSIs’ disease‐modulating effects but also the impact of targeting GGDPS in normal cells. Thus, continued exploration of this enzyme's role in normal human physiology and pathophysiology is of critical importance.

## CONFLICT OF INTEREST

The authors have no conflicts of interest to declare.
